# Massive Genomic Rearrangement Acquired in a Single Catastrophic Event during Cancer Development

**DOI:** 10.1016/j.cell.2010.11.055

**Published:** 2011-01-07

**Authors:** Philip J. Stephens, Chris D. Greenman, Beiyuan Fu, Fengtang Yang, Graham R. Bignell, Laura J. Mudie, Erin D. Pleasance, King Wai Lau, David Beare, Lucy A. Stebbings, Stuart McLaren, Meng-Lay Lin, David J. McBride, Ignacio Varela, Serena Nik-Zainal, Catherine Leroy, Mingming Jia, Andrew Menzies, Adam P. Butler, Jon W. Teague, Michael A. Quail, John Burton, Harold Swerdlow, Nigel P. Carter, Laura A. Morsberger, Christine Iacobuzio-Donahue, George A. Follows, Anthony R. Green, Adrienne M. Flanagan, Michael R. Stratton, P. Andrew Futreal, Peter J. Campbell

**Affiliations:** 1 Cancer Genome Project, Wellcome Trust Sanger Institute, Hinxton, Cambridge CB10 1SA, UK; 2 Departments of Pathology and Oncology, Johns Hopkins Medical Institutions, Baltimore, MD 21287, USA; 3 Department of Haematology, Addenbrooke's Hospital, Cambridge CB2 0QQ, UK; 4 Department of Haematology, University of Cambridge, Cambridge CB2 0XY, UK; 5 Cancer Institute, University College London, London WC1E 6BT, UK; 6 Royal National Orthopaedic Hospital, Middlesex HA7 4LP, UK; 7 Institute for Cancer Research, Sutton, Surrey SM2 5NG, UK

## Abstract

Cancer is driven by somatically acquired point mutations and chromosomal rearrangements, conventionally thought to accumulate gradually over time. Using next-generation sequencing, we characterize a phenomenon, which we term chromothripsis, whereby tens to hundreds of genomic rearrangements occur in a one-off cellular crisis. Rearrangements involving one or a few chromosomes crisscross back and forth across involved regions, generating frequent oscillations between two copy number states. These genomic hallmarks are highly improbable if rearrangements accumulate over time and instead imply that nearly all occur during a single cellular catastrophe. The stamp of chromothripsis can be seen in at least 2%–3% of all cancers, across many subtypes, and is present in ∼25% of bone cancers. We find that one, or indeed more than one, cancer-causing lesion can emerge out of the genomic crisis. This phenomenon has important implications for the origins of genomic remodeling and temporal emergence of cancer.

**PaperClip:**

## Graphical abstract

**Graphical Abstract:**

**Figure:**
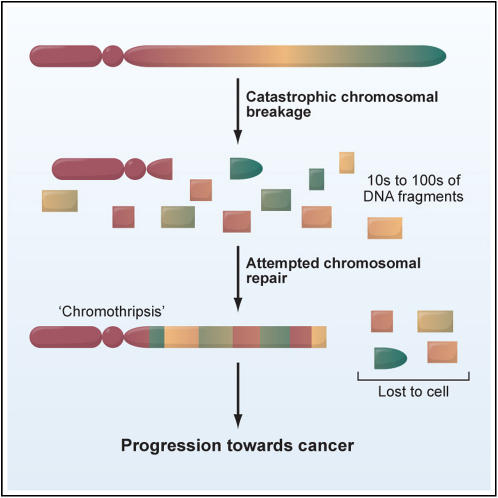

**Highlights:**

► 2%–3% cancers show 10–100 s of rearrangements localized to specific genomic regions ► Genomic features imply chromosome breaks occur in one-off crisis (“chromothripsis”) ► Found across all tumor types, especially common in bone cancers (up to 25%) ► Can generate several genomic lesions with potential to drive cancer in single event

## Introduction

The textbook model of cancer development is of progression through a series of increasingly disordered clinical and pathological phases (Stratton et al., 2009). For example, invasive colorectal cancer often emerges from an antecedent benign adenomatous polyp; cervical cancer proceeds through intraepithelial neoplasia before breaching the basement membrane; multiple myeloma frequently develops in individuals with a history of benign monoclonal plasma cell proliferation. Biologically, such stepwise clinical progression is underpinned by successive waves of clonal expansion as cells acquire the multiple genetic changes required for a fully malignant phenotype. Mutations are essentially random, occurring as independent events throughout the lifespan of an individual, potentially accelerated by exogenous carcinogens or DNA repair defects. Genetic variation generates phenotypic variation across the cells of an organ system, which are then subject to clonal selection through Darwinian competition. Variants that enhance a cell's evolutionary fitness, so-called driver mutations, promote outgrowth of that clone and progression toward cancer (Stratton et al., 2009). The prevailing dogma of cancer evolution is therefore one of “gradualism” in which acquisition of driver mutations occurs cumulatively over years to decades, resulting in incremental progression through increasingly malignant phenotypes (Jones et al., 2008).

There are, however, examples in which a more “punctuated equilibrium” evolutionary model may apply to development of cancers. Genome-wide telomere attrition in somatic cells, for example, may generate naked DNA ends that act as a nidus for on-going genomic rearrangement (Bardeesy and DePinho, 2002; O'Hagan et al., 2002; Sahin and Depinho, 2010). End-to-end chromosome fusions resulting from telomere loss can lead to spiraling cycles of dsDNA breakage, aberrant repair and further chromosomal damage in both daughter cells (Artandi et al., 2000; Gisselsson et al., 2001). Iteration of this breakage and repair process can lead to extensive genomic remodeling in multiple competing subclones in only a few cell cycles (Bignell et al., 2007). Under these scenarios, bursts of somatic mutation may accrue in relatively short periods of chronological time.

Such genomic rearrangements can drive the development of cancer through several mechanisms. They may result in copy number changes, including deletion of tumor suppressor genes and increased copy number (amplification) of genes promoting malignant cellular processes. In addition, chromosomal rearrangements can juxtapose portions of coding sequence from two genes in the same orientation, leading to oncogenic fusion genes, or bring together an intact gene with the regulatory machinery of another gene, causing dysregulated gene expression.

Here, we describe multiple cancer samples in which tens to hundreds of genomic rearrangements have been acquired in a single catastrophic event, a phenomenon we have termed chromothripsis (Greek, chromos for chromosome; thripsis, shattering into pieces). We characterize the genomic hallmarks of this process, its frequency across diverse cancers and how such cataclysmic genome disruption can promote the development of cancer.

## Results

### Localized Genomic Rearrangement in a Patient with Chronic Lymphocytic Leukemia

Advances in DNA sequencing have made it possible to identify the majority of somatically acquired genetic variants in cancer samples on a genome-wide basis (Ding et al., 2010; Mardis et al., 2009; Pleasance et al., 2010a, 2010b; Shah et al., 2009). In particular, paired-end sequencing allows discovery of genomic rearrangements (Campbell et al., 2008, 2010; Stephens et al., 2009), through sequencing both ends of 50–100 million genomic DNA fragments per sample. Alignment of the paired-end reads to the reference genome enables identification of putative genomic rearrangements.

**Figure 1:**
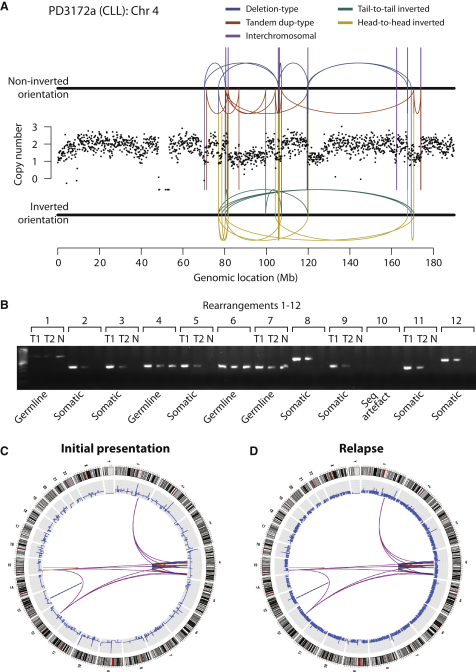
Clustered Rearrangements on Chromosome 4q in a Patient with Chronic Lymphocytic Leukemia (A) Copy number between 70 Mb and 170 Mb of the chromosome oscillates between a copy number of 1 and 2, demarcated by back-and-forth intrachromosomal rearrangements of all four possible orientations, as well as several interchromosomal rearrangements. (B) PCR gel of 12 putative genomic rearrangements identified by sequencing. PCR across the breakpoint is performed for each rearrangement on tumor DNA for samples taken at initial presentation (T1) and relapse (T2) as well as germline DNA (N). (C) Genome-wide profile of rearrangements in a sample taken before chemotherapy. Chromosomes range round the outside of the circle, copy number changes are shown by the blue line in the inner ring, and somatically acquired genomic rearrangements are shown as arcs linking the two relevant genomic points. (D) Genome-wide profile of rearrangements from the same patient 31 months later, at relapse after therapy.

In a rearrangement screen of 10 patients with chronic lymphocytic leukemia (CLL), we identified one patient who had 42 somatically acquired genomic rearrangements involving the long arm of chromosome 4 (Figures 1A and 1B and Table S1 available online). The positions of these rearrangements relative to one another and to copy number changes on chromosome 4q reveal some striking patterns. First, the rearrangements show geographic localization within the genome. Apart from a separate 13q deletion in this patient, all rearrangements are confined to chromosome 4q and focal points on chromosomes 1, 12, and 15 (Figure 1C). This is different to the patterns of genomic instability we have typically seen in breast, lung, or pancreatic cancer where rearrangements tend to be either scattered genome-wide or, if localized, are associated with substantial genomic amplification (Campbell et al., 2008; Pleasance et al., 2010b; Stephens et al., 2009). Second, the copy number profile across the chromosome arm shows many positions at which copy number changes, but these changes alternate between just two states, namely one or two copies. Analysis of allelic ratios at germline single nucleotide polymorphism (SNP) positions on chromosome 4q demonstrated that regions of copy number 1 show loss of heterozygosity, but regions of copy number 2 retain heterozygosity (data not shown). Third, the many regions of copy number 1 are not caused by simple deletions. Instead, a series of complex rearrangements spanning the involved region generate the copy number changes, as can be seen by the distribution of rearrangements falling at change-points in copy number (Figure 1A). These have both inverted and noninverted orientation, with all four orientations of intrachromosomal breakpoints represented in approximately even numbers: deletion-type (8 rearrangements), tandem duplication-type (9), head-to-head inverted (6), and tail-to-tail inverted (10). Fourth, there is pronounced clustering of breakpoints across the chromosome arm with, for example, seven rearrangements involving the 30 kb region between 77.013 Mb and 77.043 Mb, and six rearrangements in the 25 kb between 170.620 Mb and 170.645 Mb. Fifth, although the locations of DNA breaks show clustering, the two conjoined fragments of chromosome at each breakpoint are not geographically proximate. That is, there are as many rearrangements joining regions of the chromosome normally separated by tens of megabases in the germline as there are junctions between close-by regions. Sixth, there are nine rearrangements joining the long arm of chromosome 4 to other chromosomes—breakpoints on these partner chromosomes also show clustering (Figure 1C).

The sample analyzed was collected from a 62-year-old woman with CLL who had not previously received treatment. Her subsequent clinical course showed rapid deterioration, and she was treated with alemtuzumab, but unfortunately, she relapsed quickly. To assess whether the abnormalities seen in the pretreatment sample persisted in the relapsing cells or indeed showed further evolution, we sequenced a relapse specimen collected 31 months after the initial sample. All rearrangements present in the pretreatment sample were present in the later sample (Figures 1B–1D), and the striking copy number profile persisted. Furthermore, there were no new genomic rearrangements, suggesting that the process generating this complex regional remodeling had resolved before the patient was first diagnosed.

### Complex Rearrangement of Single Chromosomes Is Seen in At Least 2%–3% of All Cancers

To assess whether the unusual genomic landscape observed in the patient with CLL could be seen in other cancer samples, we analyzed high-resolution copy number profiles of 746 cancer cell lines obtained using SNP arrays (Bignell et al., 2010). Of these, 96 cell lines have at least one chromosome with >50 positions at which copy number changes (Figure S1A), many of which are caused by amplicons or other complex clusters of rearrangements. Notably, 18/746 (2.4%; 95% confidence interval, 1.5%–3.9%) cell lines have copy number profiles similar to that seen in the CLL patient, with frequent copy number changes confined to localized genomic regions rapidly alternating between one, two, or occasionally three different states (Figures S1B–S1T). Copy number changes could involve the entire chromosome (for example, SNU-C1, Figure S1G), a whole arm of a chromosome (SW982, Figure S1H), the telomeric portion of a chromosome (C32, Figure S1C), or an interstitial region of a chromosome (A172, Figure S1D). The pattern was seen in many different tumor types, including melanoma (4 cell lines), small cell lung cancer (3 cell lines), glioma (3 cell lines), hematological malignancies (2 cell lines), nonsmall cell lung cancer (1 cell line), synovial sarcoma (1 cell line), and esophageal (1 cell line), colorectal (1 cell line), renal (1 cell line), and thyroid (1 cell line) cancers. Furthermore, in segmented SNP array data from 2792 cancers, of which 80% were primary tumors (Beroukhim et al., 2010), we find evidence for chromothripsis in a similar proportion of cases (Figure S1T).

**Figure 2:**
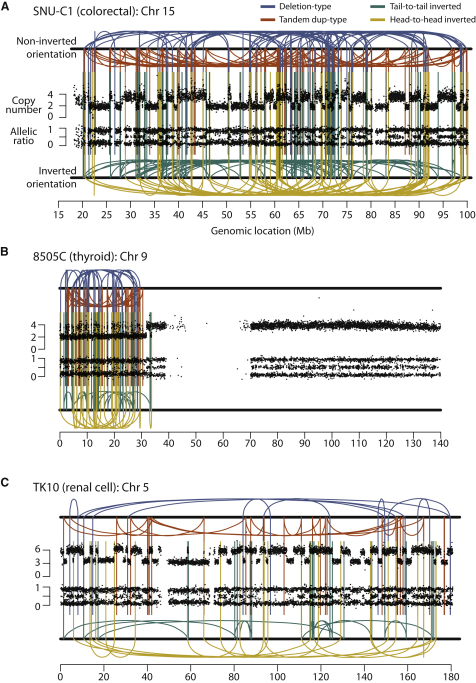
Rearrangement Screens in Three Cancer Cell Lines Showing Evidence for Chromothripsis Copy number profiles derive from SNP6 microarray data and are shown as the upper panel of points for each cell line. Allelic ratios for each SNP are shown in the lower panel of dots: homozygous SNPs cluster at allelic ratios near 0 or 1, heterozygous SNPs cluster around 0.5. Intrachromosomal rearrangements of all four possible orientations are shown, with deletion-type events as blue lines, tandem duplication-type in red, tail-to-tail inverted rearrangements in green and head-to-head inverted rearrangements in yellow. (A) SNU-C1, a cell line from a colorectal cancer, carries 239 rearrangements involving chromosome 15. (B) 8505C, a thyroid cancer cell line, has 77 rearrangements involving chromosome 9p. (C) TK10, a renal cancer cell line, has 55 rearrangements involving chromosome 5.

We selected four of these cell lines for further genomic analysis with massively parallel paired-end sequencing for rearrangements and cytogenetic studies: SNU-C1, 8505C, TK10, and SCLC-21H (described later). In SNU-C1, derived from a colorectal cancer, we identified 239 rearrangements involving chromosome 15 (Figure 2A and Table S2). From 8505C, a thyroid cancer line, we mapped 77 rearrangements involving the short arm of chromosome 9 (Figure 2B and Table S2), and for TK10, a renal cancer, 55 rearrangements involving chromosome 5 (Figure 2C and Table S2).

**Figure S2:**
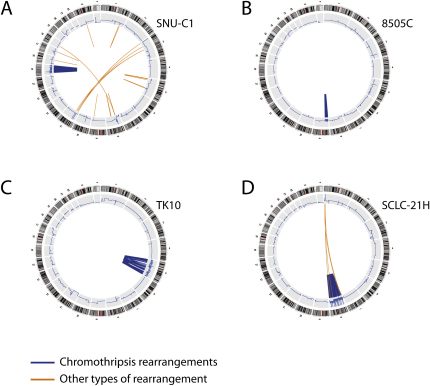
Genome-wide Rearrangements for Four Cell Lines with Chromothripsis, Related to Figure 2 (A–D) Circos plots for (A) SNU-C1, (B) 8505C, (C) TK10 and (D) SCLC-21H. Around the outside are ideograms of the chromosome, with the inner ring representing copy number segments. Chromosomal rearrangements are shown as arcs in the middle joining the two relevant regions of the genome for each rearrangement. Chromothripsis rearrangements are shown in blue and those not associated with chromothripsis in orange.

The distinctive genomic configuration observed in the CLL patient is stamped on these three cell lines. Striking geographic localization of rearrangements is evident in these samples. Although a few rearrangements were observed elsewhere in the genome (Figure S2), these are generally straightforward events such as deletions or tandem duplications and do not intersect with the regions of massive disruption shown in Figure 2. The localization is especially evident in 8505C (Figure 2B), in which rearrangements only involve the telomeric portion of chromosome 9p with sparing of the most centromeric bands of 9p and all 9q. As in the CLL patient, copy number oscillates rapidly between two states, with the lower copy number state showing loss of heterozygosity (LOH) and the higher copy number state retaining heterozygosity.

**Figure 3:**
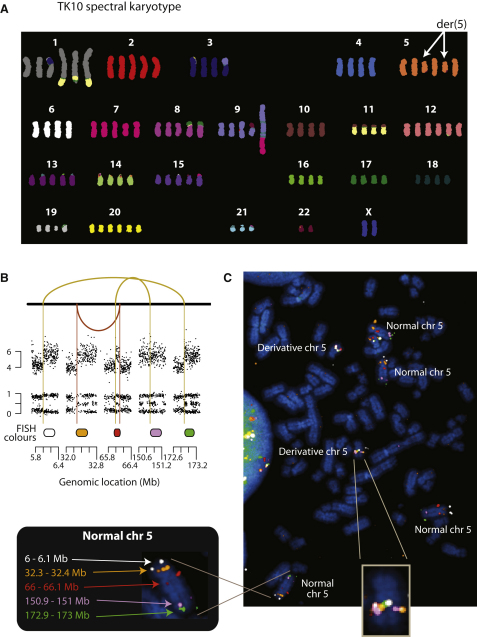
FISH Profiling of TK10 (A) The spectral karyotype of the TK10 genome. (B) Copy number profiles and genomic rearrangements for the five regions of chromosome 5 studied by multicolor FISH. (C) Multicolor FISH of TK10.

**Figure S3:**
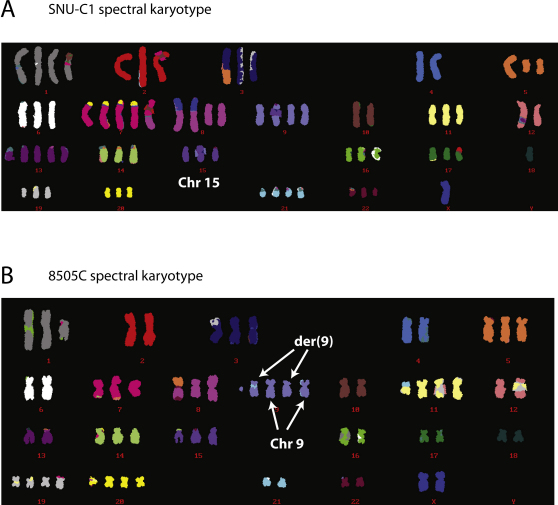
Spectral Karyotypes for (A) 8505C and (B) SNU-C1, Related to Figure 3

One question that arises is whether the rearrangements are all found on a single parental copy of the chromosome or whether both copies are involved. We therefore performed spectral karyotyping on the three cell lines (Figure 3A and Figure S3). TK10, a hyperdiploid line, carries six copies of chromosome 5. Consistent with the observed copy number profile alternating between states of copy number 4 with LOH and copy number 6 with heterozygosity, the karyotype showed four grossly normal copies of chromosome 5 and two smaller derivative chromosomes. Similarly, in 8505C, two copies of chromosome 9 showed distinctly foreshortened p arms alongside two cytogenetically normal chromosomes. None of the three karyotypes indicated translocations involving the respective derivative chromosomes, confirming the impression from the paired-end sequencing data that the genomic remodeling of these regions was entirely intrachromosomal. Cytogenetic changes were consistently seen across all cells examined.

The spectral karyotypes suggest that the rearrangements involve a single parental copy of the chromosome. To demonstrate this further, we designed FISH probes to five widely dispersed regions of chromosome 5 at copy number 6 in TK10 (Figure 3B). From the paired-end sequencing, we predicted that the two regions at 6 Mb and 172 Mb would be joined by a head-to-head inverted rearrangement, and the three regions at 32 Mb, 66 Mb, and 150 Mb would be joined by another head-to-head inverted rearrangement and a tandem duplication-type rearrangement. These FISH probes, labeled with different dyes, were hybridized to TK10 cells (Figure 3C). As expected, there were four copies of chromosome 5 per cell showing the correct genomic orientation and distribution of the five probes. In addition, each cell carried two copies of a derivative 5 chromosome in which all five probes were closely juxtaposed, as predicted by the sequencing data. These patterns were seen identically across all cells examined.

Taken together, these data suggest that at least 2%–3% of all cancers show evidence for massive remodeling of a single chromosome, involving tens to hundreds of genomic rearrangements. The consistency of cytogenetic findings across the many cells examined implies that the clustering of genomic breakpoints cannot be explained by multiple, parallel rearrangements in different subclones. In the lines studied here, the genomic remodeling occurred when there were just the two parental copies of the relevant chromosome, preceding chromosomal duplication events. This explains why copy number states alternate between heterozygous and LOH and why more than one copy of the derivative chromosome is present.

### Chromothripsis Is Particularly Common in Bone Cancers and Can Involve More Than One Chromosome

Alongside the rearrangement screen in CLL, we performed rearrangement screens in primary tumor samples from 20 patients with bone cancer, including 9 with osteosarcoma and 11 with chordoma, a rare type of cancer arising in the spinal column. Strikingly, five of these patients (25%; 95% confidence interval, 10%–49%), three with osteosarcoma and two with chordoma, also show large numbers of clustered rearrangements with the hallmarks of chromothripsis.

**Figure 4:**
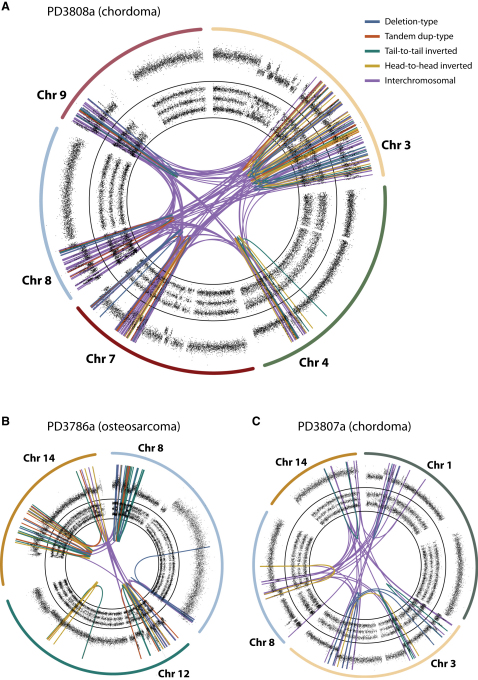
Chromothripsis Involving More Than One Chromosome in Primary Samples from Patients with Bone Cancer For each case, the relevant chromosomes are shown with SNP6 microarray copy number profiles in the outer ring, allelic ratios in the inner ring, and somatically acquired genomic rearrangements shown as arcs in the center. (A) PD3808a, from a chordoma, shows 147 rearrangements interlinking chromosomes 3q, 4q, 7q, 8p, and 9p. (B) PD3786a, an osteosarcoma sample, carries 88 rearrangements involving chromosome 8, 12, and 14. (C) PD3807a, another chordoma sample, has 38 rearrangements involving chromosomes 1p, 3, 8, and 14.

**Figure S4:**
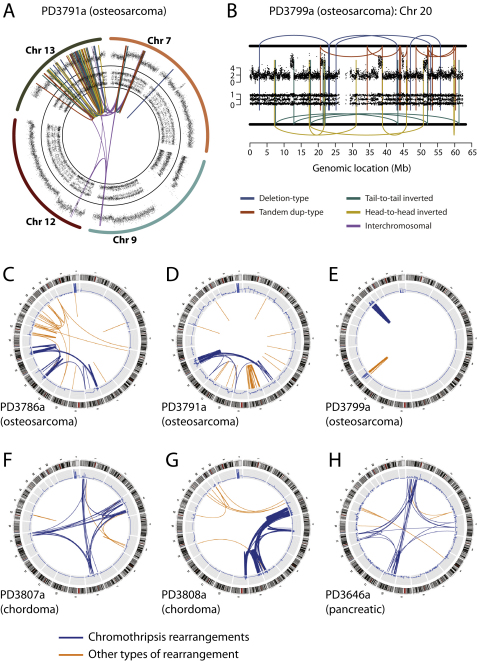
Chromothripsis Involving Several Chromosomes, Related to Figure 4 (A) PD3791a, with osteosarcoma, shows 86 rearrangements involving chromosomes 7, 9, 12 and 13, with SNP6 microarray copy number profiles in the outer ring, allelic ratios in the inner ring and somatically acquired genomic rearrangements shown as arcs in the centre. (B) PD3799a, also from a patient with osteosarcoma, shows 28 rearrangements involving chromosome 20. (C–H) Circos plots for (C) PD3786a, (D) PD3791a, (E) PD3799a, (F) PD3807a, (G) PD3808a and (H) PD3646a. Around the outside are ideograms of the chromosome, with the inner ring representing copy number segments. Chromosomal rearrangements are shown as arcs in the middle joining the two relevant regions of the genome for each rearrangement. Chromothripsis rearrangements are shown in blue and those not associated with chromothripsis in orange.

In four of these five bone tumors, rearrangements affect localized regions of several chromosomes (Figure 4, Figure S4, Table S3, and Table S4). For example, we identified 147 somatically acquired genomic rearrangements in a chordoma sample, PD3808a, involving and linking together well-circumscribed regions of chromosomes 3q, 4q, 7q, 8p, and 9p (Figure 4A). Analogous to chromothripsis involving single chromosomes, copy number in each of these chromosomal regions cycles between two different states with retention of heterozygosity in the higher copy number state. Of the 147 rearrangements, 49 are intrachromosomal and show the same back-and-forth mixture of inverted and noninverted rearrangements described above. The numerous interchromosomal rearrangements link the various disrupted regions together, implying that the resulting genomic structure is a complex medley of fragments from different chromosomes jumbled together.

In samples from three patients with osteosarcoma, PD3786a (Figure 4B), PD3791a (Figure S4A), and PD3799a (Figure S4B), we identified 88, 86 and 24 rearrangements respectively with similar overall patterns of copy number change and rearrangement. PD3807a, another chordoma sample, also had 38 rearrangements interlinking well-defined regions of four chromosomes (Figure 4C). Clinically, the patients ranged in age from 9 to 64 years and four of the samples were from resections of treatment-naive primary tumors, whereas one of the patients (PD3786a) had previously received neoadjuvant chemotherapy. In 1 of 13 pancreatic cancers we previously sequenced (Campbell et al., 2010), we identified 41 rearrangements involving chromosomes 1, 4, 10, and 14 with the hallmarks of chromothripsis (Figure S4H), suggesting that involvement of multiple chromosomes by this process is not restricted to bone tumors.

### The Vast Majority of Chromothripsis Rearrangements Occur in a Single Catastrophic Event

**Figure 5:**
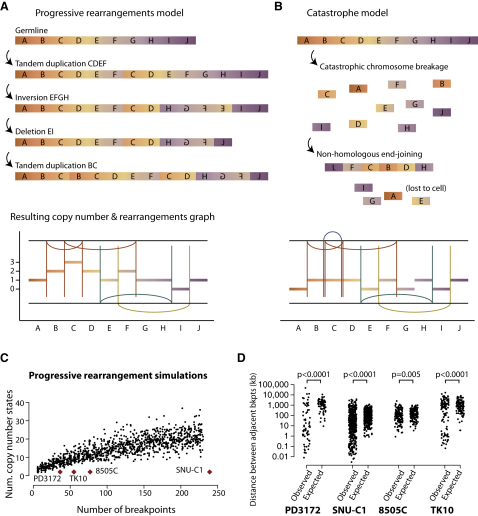
Genomic Features of Chromothripsis Suggest that Most Rearrangements Occur in a Single Catastrophic Event (A) Example of a sequence of progressive rearrangements disrupting a model chromosome. The chromosomal configuration after each rearrangement is shown, together with the copy number and rearrangement plot that would result (in the style of Figure 2). (B) Example of how a chromosomal catastrophe might break the chromosome into many pieces that are then stitched back together haphazardly. (C) One thousand Monte Carlo simulations (black points) performed under the assumption that rearrangements accumulate progressively over time show the number of copy number states seen in the resultant derivative chromosome. Samples with chromothripsis, shown as red diamonds, fall well outside this spectrum. (D) Observed distances between adjacent breakpoints for each sample are shown beside the expected distribution if breaks occurred in entirely random locations.

There are two potential models for how such complex restructuring of a chromosome could develop. Under the progressive rearrangement model, the rearrangements occur sequentially and independently of one another over many cell cycles, leading to increasingly disordered genomic structure (Figure 5A). This is the conventional view of how most complex regional clusters of rearrangements evolve, especially genomic amplification. Localization results either from rearrangement targeting a specific cancer gene or through regional abnormalities driving recurrent DNA breakage. The second model to explain the distinctive genomic structures described here is that the overwhelming majority of rearrangements occur in a single catastrophic event. In this scenario, the chromosome or chromosomal region shatters into tens to hundreds of pieces, some (but not all) of which are then stitched together by the DNA repair machinery in a mosaic patchwork of genomic fragments (Figure 5B).

Several characteristics of the patterns we observe here make the progressive rearrangement model difficult to sustain, and give support to the catastrophe model. The first observation is that the number of copy number states observed in the final configuration of the chromosome is restricted to two (occasionally three). With sequential, independent rearrangements, the number of different states observed would be expected to increase as the number of breakpoints rises (Figure 5A). Tandem duplications increase copy number and, because many of the observed rearrangements with a tandem duplication pattern in these samples overlap with one another, we would anticipate a number of segments to have been sequentially amplified several-fold under the progressive rearrangements model. Although deletion events would tend to counteract increases in copy number, the chances of these two processes being so balanced as to generate only two copy number states fall rapidly as the number of rearrangements increases. To demonstrate this, we performed Monte Carlo simulations of the progressive rearrangement model. Rearrangements were randomly sampled from the set of breakpoints found in SNU-C1, the resulting chromosome structure calculated, and the process repeated to generate different numbers of rearrangements (Figure 5C). As predicted, with increasing numbers of rearrangements, the observed number of different copy number states also rises. The observed profiles of the three cell lines and the CLL patient sit well outside the spectrum observed under simulations of the progressive rearrangement model.

In contrast, the catastrophe model predicts exactly two copy number states. Those fragments that are retained in the eventual derivative chromosome will have the higher copy number state; those that are lost to the cell will be in the lower copy number state (Figure 5B).

The second problem for the progressive rearrangements model is the retention of heterozygosity in regions with higher copy number. Once lost, heterozygosity cannot generally be regained. For example, the region around 66 Mb of chromosome 15 of SNU-C1 is heterozygous, but is encompassed in the span of no fewer than 21 rearrangements with the orientation of deletions, as well as 20 tandem duplication-type and 52 inverted rearrangements (Figure 2A). Under the progressive rearrangement model, a deletion that occurred early in the sequence of rearrangements would permanently remove heterozygosity between the breakpoints. Thus, deleting events can only occur late in the succession of rearrangements, once regions of retained heterozygosity have either been switched out of the region by inversion or copied by tandem duplication. When extended across all 239 rearrangements involving chromosome 15, there is major difficulty constructing a sequence of progressive rearrangements that would spare the heterozygosity found in over 20 separate segments. In contrast, alternating regions of heterozygosity and LOH is the natural consequence of the catastrophe model. With a normal parental chromosome and one shattered into many pieces, any fragment that is retained in the eventual derivative chromosome will be heterozygous; those that are lost to the cell will result in LOH in those regions (Figure 5B).

**Figure S5:**
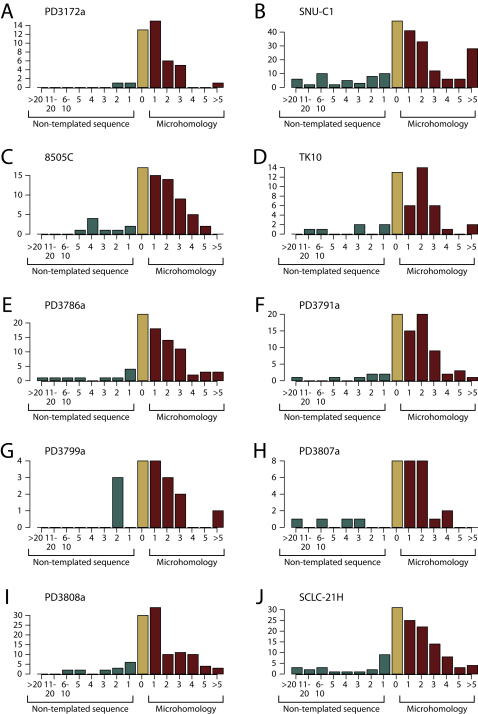
Signatures of DNA Repair at the Breakpoint, Related to Figure 5 Patterns of microhomology (red), non-templated sequence (teal) or direct end-joining (yellow) in the chromothripsis rearrangements for each sample. The x axis shows the number of bases of microhomology (right of 0) or non-templated sequence (left of 0) for each rearrangement. The y axis shows the number of rearrangements in the sample showing that pattern of microhomology or non-templated sequence.

A third feature arguing against the progressive rearrangement model is that breakpoints show significantly more clustering along the chromosome or chromosome arm than expected by chance (Figure 5D). A clean break across double-stranded DNA (dsDNA) generates two naked ends of which none, one or two may subsequently be repaired. Some of the clustering represents erroneous repair of both sides of a dsDNA break (see Figure 5B, for example). The extent of clustering observed in breakpoint locations, however, is much greater than explicable by this means alone. This presents some difficulties for the progressive rearrangements model because such nonrandom distribution of independently generated breaks would imply extensive regional variation in chromosomal fragility. Specific regions of increased propensity to rearrangement have been documented (Bignell et al., 2010), but not to the extent observed here. Under a catastrophe model, clustering among the prolific numbers of DNA breaks would perhaps be expected, depending on the process causing the DNA damage and repair. The limited overlap between sequences at the breakpoint junction suggests that the major mechanisms of DNA repair here are microhomology-mediated break repair and/or nonhomologous end-joining rather than homologous recombination (Figure S5).

In conclusion, several distinctive genomic features imply that a major catastrophic event underpins the massive, but localized, genomic rearrangement in these samples. These arguments extend to cancers where we have observed involvement of several different chromosomes. We do not argue that absolutely every rearrangement was generated in one event—indeed, a later partial duplication of the derivative chromosome is likely to explain why some samples (such as C32, Figure S2C) oscillate across three copy number states rather than two. However, the majority of rearrangements seen in these examples almost certainly occurred in a single event.

### Chromothripsis Can Generate Genomic Consequences that Promote Cancer Development

A cell suffering tens to hundreds of DNA breaks in a single cataclysmic event would be expected to undergo apoptosis. That a cell can survive such an insult and progress to become cancerous suggests that the extensive remodeling of the genome may confer significant selective advantage to that clone. To explore this possibility, we analyzed the genomic data for evidence of changes that might promote the development of cancer.

**Figure 6:**
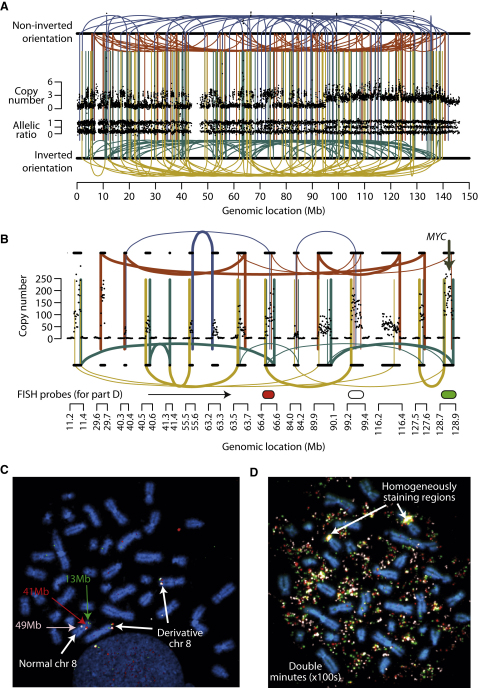
Generation of a Double Minute Chromosome Containing *MYC* by Chromothripsis in a Small Cell Lung Cancer Cell Line, SCLC-21H (A) Copy number profile, allelic ratio, and rearrangements of chromosome 8. (B) Copy number data from the rearrangement screen shows 15 discrete regions of chromosome 8 that are massively amplified, with 50–200 copies per cell. Each amplified region is demarcated by rearrangements linking to other heavily amplified segments (thick lines), with evidence for later internal rearrangements also found (thin lines). (C) Three color FISH for three regions of chromosome 8 (predicted to be linked by the rearrangement data, but not amplified; green, 13 Mb; red, 41 Mb; pale pink, 49 Mb). (D) FISH for three heavily amplified regions. The locations of the probes are shown in Figure 6B (red, 66.5 Mb; white, 99.3 Mb; green, 128.8 Mb).

One small cell lung cancer cell line, SCLC-21H, demonstrates massive numbers of copy number changes on chromosome 8, mostly with the typical appearances of chromothripsis (Figure S2A). Interestingly, however, the SNP array data suggest that some segments of the chromosome might be heavily amplified. We mapped 170 breakpoints, all involving chromosome 8 and showing the expected patterns of rearrangements described above (Figure 6A and Table S2). Whereas most of the chromosome oscillates among low copy number states, there are 15 discrete segments of the chromosome present at markedly increased copy number, ranging from 50 to 200 copies per cell (Figure 6B). One of these segments contains the *MYC* oncogene, amplified in 10%–20% of small cell lung cancers (Sher et al., 2008). The rearrangement data demonstrate that the 15 regions are interwoven by a series of rearrangements, many of which demarcate the starts and ends of the massively amplified segments. Strikingly, we found no evidence for breakpoints linking these massively amplified regions to the other, nonamplified but rearranged, regions of chromosome 8.

One potential mechanism for these findings is that at some stage while the cancer was evolving, chromosome 8 shattered into hundreds of pieces. Many of these were stitched together into a derivative chromosome 8, but 15 other fragments were joined to create a double minute chromosome of ∼1.1Mb in size (thick lines, Figure 6B). Containing *MYC*, it was of considerable selective advantage for daughter cells to carry extra copies of the double minute, and through further internal rearrangements (thin lines, Figure 6B) and overreplication, the massive amplification evolved.

**Figure S6:**
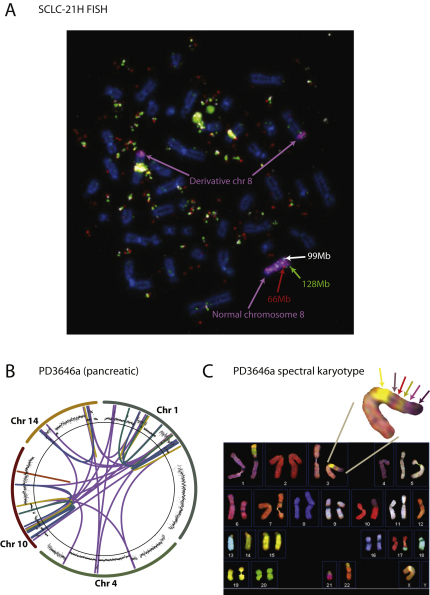
Marker Chromosomes Created by Chromothripsis, Related to Figure 6 (A) Multicolor FISH for the three heavily amplified probes in SCLC-21H (shown in Figure 6B) together with whole chromosome paint for chromosome 8 (purple). The normal chromosome 8 has the three probes in the correct orientation, whereas the probes do not hybridize to the two derivative chromosomes. (B) A rearrangement screen from a pancreatic cancer, PD3646a, previously published (Campbell et al., 2010), shows 41 rearrangements involving chromosomes 1, 4, 10 and 14. Copy number profiles for the relevant chromosomes are in the outer ring with somatically acquired genomic rearrangements shown as arcs in the centre. (C) Spectral karyotype for PD3646a shows a striped marker chromosome, with at least 6 cytogenetically visible segments arising from multiple chromosomes. The bright yellow signal corresponds to paint from chromosome 22, but the other stripes are characteristic of signals from chromosomes 1, 4, 10 and 14.

To assess this hypothesis, we performed multicolor FISH. First, we probed three nonamplified segments of chromosome 8 that the sequencing suggested were joined together through a head-to-head inverted rearrangement and a tandem duplication-type rearrangement. This revealed a single normal copy of chromosome 8 with the probes hybridized in the expected orientation and distance apart, and two derivative 8 chromosomes with the three probes closely juxtaposed (Figure 6C). Thus, the cells contain a cytogenetically normal chromosome 8 and a derivative chromosome 8 generated by chromothripsis that has subsequently undergone chromosomal duplication. Second, we probed three of the chromosome 8 regions that were heavily amplified (Figure 6D). This demonstrated huge numbers of extrachromosomal copies of the segments, with the probes closely abutting. In addition, there were two homogeneously staining regions identified by the probes, consistent with chromosomal integration of the double minutes. Probes for the double minute chromosomes were found in the correct orientation on the normal chromosome 8, but were absent from the two copies of the derivative chromosome 8 (Figure S6A). Taken together, these findings are consistent with the model that the catastrophic shattering of chromosome 8 has facilitated the creation of a double minute chromosome, which, in this example, containing *MYC*, acts as a substrate for amplification, evolutionary selection and progression toward cancer.

Chromothripsis may lead to the generation of other forms of marker chromosome also. We studied the spectral karyotype of the pancreatic cancer sample with evidence for chromothripsis involving multiple chromosomes (Figures S6B and S6C). Even with the low resolution of SKY, a chromosome arm with at least six cytogenetically visible stripes could be seen, indicating that the many interchromosomal rearrangements have intertwined segments from multiple different chromosomes into a distinctive marker chromosome.

**Figure 7:**
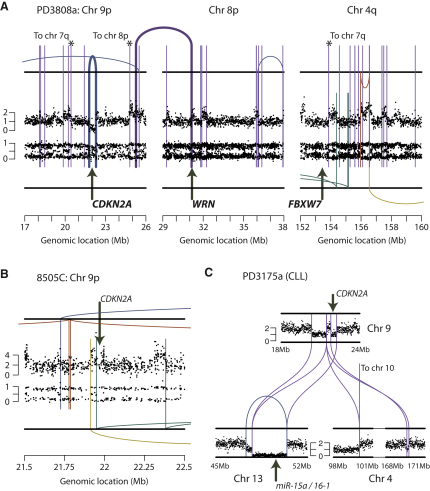
Loss of Tumor Suppressor Genes through Chromothripsis (A) PD3808a, the chordoma sample shown in Figure 4A, shows clustered chromothripsis rearrangements around *CDKN2A*, leading to a loss of one copy of this tumor suppressor gene. The other copy is also lost, through a deletion, which presumably occurred on the other parental copy of chromosome 9p at a separate time-point (thick line). The same cluster of chromothripsis rearrangements causes loss of a second tumor suppressor gene, *FBXW7*, on chromosome 4q and a third cancer gene, *WRN*, on chromosome 8p. (B) Chromothripsis has also led to loss of one copy of *CDKN2A* in the thyroid cancer cell line, 8505C. (C) Loss of two tumor suppressor genes, *CDKN2A* and the microRNA cluster *miR-15a/16-1*, by clustered rearrangements involving chromosomes 4, 9, and 13 in a patient with CLL, PD3175a.

A second potential mechanism by which chromothripsis could generate cancer-causing genomic changes is through loss or disruption of tumor suppressor genes. In the chordoma, PD3808a, the *CDKN2A* gene is homozygously deleted (Figure 7A), with one of the copies probably lost through chromothripsis. The two rearrangements demarcating the copy number change from 2 to 1 around *CDKN2A* (marked with ^∗^ in Figure 7A) appear to be part of the network of interchromosomal rearrangements interlinking regions from chromosomes 3q, 4q, 7q, 8p, and 9p seen in Figure 4A. This argues that loss of this copy of the gene occurred during chromothripsis, although it is formally possible that an independent deletion of *CDKN2A* might have occurred before chromothripsis. The second copy of the gene was lost through a focal deletion on the other parental chromosome, which presumably occurred as a temporally separate event (thick blue line, Figure 7A).

With so many rearrangements generated in a single genomic crisis, it is feasible that more than one cancer-causing lesion could occur in the same event. In addition to the loss of *CDKN2A* described above, the chordoma sample PD3808a had a rearrangement that directly disrupted *WRN*, linking the 3′ portion of this gene on chromosome 8 to an intergenic region on 9p just downstream of *CDKN2A* (thick purple line, Figure 7A). *WRN* is a cancer gene in which germline mutation causes Werner syndrome, a condition associated with markedly increased risk of bone tumors, and in which somatic inactivating mutations have been documented in renal cancer (Dalgliesh et al., 2010). This same patient also lost a copy of *FBXW7* on chromosome 4q (Figure 7A). The rearrangements around this gene link to chromosomes 3q, 7q, 8p, 9p, and elsewhere on 4q similar to those near *CDKN2A* and *WRN*, suggesting that loss of *FBXW7* occurred during the same chromothripsis event. *FBXW7* is inactivated in ∼6% of all cancers across many subtypes (Akhoondi et al., 2007; Kemp et al., 2005; Maser et al., 2007). Inactivation is frequently heterozygous, supported by functional data suggesting it may be a haploinsufficient tumor suppressor gene (Kemp et al., 2005; Mao et al., 2004). Thus, the single catastrophic event inducing chromothripsis in this patient has resulted in disruption of three tumor suppressor genes.

A number of other known cancer genes were affected by rearrangements across the samples described here (Table S5), including *ARID1A* in PD3807a (chordoma). In 8505C, the chromothripsis involving chromosome 9p has led to loss of one copy of *CDKN2A* (Figure 7B); the other carries a deletion of the first exon of the gene. We also identified a second patient with CLL who showed evidence for loss of two tumor suppressor genes in a cluster of rearrangements involving chromosomes 4, 9, and 13 (Figure 7C). Here, single copies of both *CDKN2A* and *miR-15a/16-1*, the microRNA cluster deleted in >50% of CLL patients (Cimmino et al., 2005), were lost through interchromosomal rearrangements, whereas the other copy of the microRNA cluster was deleted in a presumably separate event (blue line, Figure 7C).

Theoretically, chromothripsis rearrangements could juxtapose coding portions of two genes in the same orientation with an open reading frame, producing a potentially oncogenic fusion gene. Among chromothripsis rearrangements, we found 17 that could potentially create novel in-frame fusions (Table S5). None generates a classic cancer-associated fusion gene, such as *BCR-ABL1* or *EWS-FLI1*, and the proportion of rearrangements generating novel in-frame fusions is similar to that observed for other types of rearrangements (Campbell et al., 2010; Stephens et al., 2009). This suggests that most are coincidental “passenger” events, unlikely to drive cancer development.

**Figure S7:**
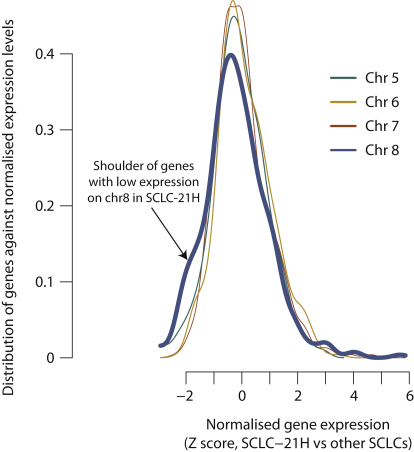
Comparison of Gene Expression Profiles from the Chromothripsis Chromosome for SCLC-21H against Intact Chromosomes, Related to Figure 7 Comparison of gene expression profiles from the chromothripsis chromosome (chr 8, blue line) for SCLC-21H against intact chromosomes (chr 5, 6 and 7; green, yellow and red lines respectively).The expression level of each gene is expressed as a Z score compared against the set of other SCLC cell lines (x axis), and it is the density / distribution of these Z scores that is plotted (y axis).

Such dramatic restructuring of a genome will disrupt both coding sequences directly and the linkage between coding exons and regulatory elements of very many genes. We explored whether expression profiles of genes from chromosomes affected by chromothripsis differed from those of intact chromosomes. For SCLC-21H, genes from chromosomes that were not affected by chromothripsis showed an approximately normal distribution of expression levels relative to their expression in other SCLC cell lines (Figure S7), as expected. On chromosome 8, however, affected by chromothripsis, expression levels were decreased in ∼5% of genes in SCLC-21H relative to their expression in other SCLC cell lines (chromosome 7 versus chromosome 8, p = 0.001; chromosome 6 versus chromosome 8, p < 0.0001). Similar differences were observed for SNU-C1, in which chromothripsis affected chromosome 15 (chromosome 14 versus chromosome 15, p = 0.02; chromosome 13 versus chromosome 15, p < 0.0001).

Taken together, these data exemplify the mechanisms by which chromothripsis can promote the development of cancer. In particular, more than one cancer-causing lesion can arise from a single catastrophe, and the chaotic genomic architecture that results can inactivate or disrupt the transcription of many more genes.

## Discussion

Here we describe a quite remarkable phenomenon whereby tens to hundreds of chromosomal rearrangements involving localized genomic regions can be acquired in an apparently one-off cellular catastrophe. Astoundingly, not only can a cell actually survive this crisis, it can emerge with a genomic landscape that confers a significant selective advantage to the clone, promoting the evolution toward cancer. Such an event appears to have occurred in 2%–3% of all cancers, across many subtypes, and may be particularly frequent in bone cancers.

There are few documented examples of how catastrophic genomic change affects evolutionary processes. Reassortment of influenza virus genomes can lead to entirely novel strains with considerable pandemic potential (Neumann et al., 2009). In eukaryotes, “showers” of several point mutations in a localized genomic region in a single cell cycle have been described in murine models (Wang et al., 2007), with similar arguments extended to clustered mutations in humans with germline genetic diseases (Chen et al., 2009). We would predict that in the case of chromothripsis, the overwhelming majority of cells suffering such spectacular genomic damage would either die or acquire more detrimental than advantageous variants. However, very rarely, a cell might acquire one or more cancer-causing lesions from such an event and this clone would then have taken a considerable leap along the road to cancer. There would still be the need for additional mutations in cancer genes, exemplified by the second hits in *CDKN2A* seen in the chordoma and thyroid cancer samples (Figure 7), but we might anticipate the emergent tumor having shorter latency.

What causes such dramatic damage to the genome? The distinctive signature of the process gives some clues. The genomic regions involved in each example are sharply circumscribed, whether it be a whole chromosome, a chromosome arm or a region of just a few megabases within a chromosomal band. It seems likely that the insult occurs while the chromosomes are condensed for mitosis. During interphase, chromosomes are relaxed with long loops of DNA winding through the nucleus: although given chromosomes occupy general nuclear territories, these tend to be loosely defined and nonexclusive (Misteli, 2007). DNA damage acquired in interphase would seem unlikely to exhibit such intense clustering of breaks within such well-circumscribed genomic regions. The existence of rearrangements involving both sides of a DNA break, the potential to create both a derivative chromosome and a double minute chromosome in the same event and the seeming near-randomness of which fragment is joined to which fragment suggest that literally hundreds of shards of genomic DNA circulate unfettered in the nucleus during the catastrophe, that the DNA repair machinery is pasting them together in a helter-skelter tumult of activity.

The agent of this physical chromosomal damage is unknown. One appealing possibility is ionizing radiation. Well-known to induce dsDNA breaks, a pulse of ionizing radiation could cut a swathe through a condensed chromosome and, depending on whether the angle of the path relative to the long axis of the chromosome is transverse, oblique or longitudinal, generate breaks involving a band, an arm or the whole chromosome. Such a model could potentially be tested by in vitro studies of cells surviving irradiation and by analysis of cancer genomes from patients with prior environmental or therapeutic radiation exposure.

Another intriguing possibility is that the breakage-fusion-bridge cycle associated with telomere attrition could induce the damage, especially because most examples of chromothripsis observed here involve regions extending to the telomeres (Figure S1). End-to-end chromosome fusions are a cytogenetic hallmark of telomere loss (Artandi et al., 2000; Gisselsson et al., 2001; O'Hagan et al., 2002), and the two centromeres of such dicentric chromosomes are pulled to opposite daughter cells during anaphase, forming a so-called anaphase bridge (Bignell et al., 2007; McClintock, 1941; Sahin and Depinho, 2010). It is unclear how these bridges are resolved, but they appear to induce the formation of nuclear buds and micronuclei containing fragmented DNA in the daughter cells (Pampalona et al., 2010). It is therefore conceivable that the dramatic stretching and pinching of the chromosome bridge during the final stages of cytokinesis could be associated with catastrophic, but localized, genomic damage. If this hypothesis is true, cancer genomes from genetically engineered mouse models of telomerase deficiency (Artandi et al., 2000; Maser et al., 2007; O'Hagan et al., 2002) may demonstrate similar patterns of genomic rearrangement to those observed here.

Whatever the mechanism of damage, the consequences are profound. Faced with hundreds of DNA breaks, the cell's DNA repair machinery attempts to rescue the genome. The resultant hodgepodge bears little resemblance to its original structure, and the genomic disruption has wholesale and potentially oncogenic effects.

## Experimental Procedures

### Samples

Rearrangement screens were performed on genomic DNA from 10 patients with chronic B cell lymphocytic leukemia attending Addenbrooke's Hospital, Cambridge, UK. Screens were also performed on genomic DNA samples from 20 patients with bone cancer (9 osteosarcoma, 11 chordoma) collected at the Royal National Orthopaedic Hospital, Middlesex, UK. From all 30 samples, we had germline DNA available. Informed consent was obtained from all patients or guardians and samples were collected and analyzed with approval from relevant Ethics Committees. The cell line set has previously been described (Bignell et al., 2010), and for the four samples presented here, germline DNA was not available.

### Massively Parallel Sequencing

The protocols for massively parallel, paired-end sequencing to identify somatically acquired genomic rearrangements in cancer samples have been described in detail elsewhere (Campbell et al., 2008; Quail et al., 2008; Stephens et al., 2009). In brief, 5 μg of genomic DNA from the tumor sample was sheared to fragments 400–500 base pairs (bp) in size. Sequencing of 37 bp from either end was performed on the Illumina Genome Analyzer II platform. Reads were aligned to the reference human genome (NCBI build 36) using MAQ (Li et al., 2008). Putative genomic rearrangements were screened by PCR across the breakpoint in tumor DNA samples and, where available, germline DNA.

### SNP Array Analyses

Tumor DNA samples from the 20 patients with bone cancer and the cell line set were also analyzed by Affymetrix SNP6 microarrays, as described (Bignell et al., 2010). Copy number and allelic ratio profiles were statistically processed using the PICNIC algorithm (Greenman et al., 2010).

### Multiplex-Fluorescence In Situ Hybridization

Human 24 color M-FISH paint was made essentially following the “pooling” strategy described (Geigl et al., 2006). Briefly, individual human chromosome-specific DOP-PCR products were grouped into five re-amplifiable pools based on the fluorescence label and subsequently labeled with biotin-16-dUTP, Texas Red-12-dUTP, Cy3-, Cy5-dUTP, and Green-dUTP. Labeled DNA was precipitated with human Cot-1 DNA. Where used, human fosmid clones were selected according to their positions in the hg17 reference assembly. Biotin-labeled probe was detected with one layer of Cy5.5-conjugated mouse anti-biotin. Metaphases were examined with either a Leica DM5000 or a Zeiss AxioIamger D1 fluorescence microscope.

### Statistical Analysis

Simulations of the progressive rearrangement model were performed 1000 times using the 239 rearrangements involving chromosome 15 identified in SNU-C1. Starting with a wild-type chromosome 15, rearrangements were randomly selected without replacement from the set of 239 events. At each step, the relevant rearrangement was applied to the current configuration of the chromosome: for example, a deletion-type rearrangement would lead to loss of intervening sequence between the breakpoints. Where the selected rearrangement was impossible (that is, one breakpoint occurred in a region already lost to the chromosome in that simulation), it was discarded and another selected. Where more than one copy of the breakpoint location existed in the current configuration (for example, the region had undergone tandem duplication in a previous rearrangement), which copy of the breakpoint location to use was chosen randomly. The number of unique copy number states across the chromosome was monitored for each simulation.

To test whether the locations of genomic breakpoints showed more clustering than expected by chance, Kolmogorov-Smirnov tests were used to compare the observed distribution of distances between adjacent breaks and that expected under the null hypothesis (exponential distribution).

For analysis of expression levels of genes from chromothripsis chromosomes compared to intact chromosomes, the expression levels of every gene on the relevant chromosomes were converted to *Z*-scores using the expression levels for other cell lines from the same tumor type. The distribution of *Z*-scores for the chromothripsis chromosome was then compared to the distribution for other chromosomes by the Kolmogorov-Smirnov test.

The circle plots were generated with Circos (Krzywinski et al., 2009).

## Accession Numbers

Genome sequence data have been deposited at the European Genome-Phenome Archive (http://www.ebi.ac.uk/ega/, hosted by the EBI) with accession number EGAD00001000002.

## Supplemental Information

Table S1. Rearrangements Identified by Massively Parallel, Paired-End Sequencing in Three Samples from Two CLL Cases: PD3172a, PD3172c, and PD3175a, Related to Figure 1

Table S2. Rearrangements Identified by Massively Parallel, Paired-End Sequencing in Four Cell Lines: SNU-C1, 8505C, TK10, and SCLC-21H, Related to Figure 2

Table S3. Rearrangements Identified by Massively Parallel, Paired-End Sequencing in Two Chordoma Samples: PD3807a and PD3808a, Related to Figure 4

Table S4. Rearrangements Identified by Massively Parallel, Paired-End Sequencing in Three Osteosarcoma Samples: PD3786a, PD3791a, and PD3799a, Related to Figure 4

Table S5. Fusion Genes and Known Cancer Genes with Breaks among Chromothripsis Rearrangements, Related to Figure 7 Known cancer genes are colored according to their mechanism of action. Red, translocation partner; green, activation by mutation (dominant oncogene); blue, inactivation by mutation (tumor suppressor gene).

Document S1. Analysis of SNP Array Copy Number Profiles from 746 Cancer Cell Lines for Appearances Suggestive of Chromothripsis, Related to Figure 1 (A) The graph charts the number of copy number changes in the most rearranged chromosome against the ratio of the number of segments with the commonest copy number state to the number in the second-commonest state. Cell lines with the appearances of chromothripsis are shown as orange points. (B–S) Copy number profiles of the relevant chromosome for the 18 cell lines identified to have changes suggestive of chromothripsis. The top panel for each cell line shows the copy number profile across the chromosome, the middle row shows the probability of a change in copy number state at each position and the lower panel shows the allelic ratio. Homozygous SNPs cluster at allelic ratios near 0 or 1, heterozygous SNPs cluster around 0.5. (T) Segmented copy number profiles with evidence of chromothripsis from representative samples in the ‘Tumorscape’ database from the Broad Institute (Beroukhim et al., 2010).

Document S2. Article Plus Supplemental Information
