# Supplementary material for: Massive Genomic Rearrangement Acquired in a Single Catastrophic Event during Cancer Development
Source: Cell. 2011 Jan 7;144(1):27–40. doi: 10.1016/j.cell.2010.11.055 (PMC3065307; doi:10.1016/j.cell.2010.11.055)
Supplement: Document S1. Analysis of SNP Array Copy Number Profiles from 746 Cancer Cell Lines for Appearances Suggestive of Chromothripsis, Related to Figure 1 — (A) The graph charts the number of copy number changes in the most rearranged chromosome against the ratio of the number of segments with the commonest copy number state to the number in the second-commonest state. Cell lines with the appearances of chromothripsis are shown as orange points. (B–S) Copy number profiles of the relevant chromosome for the 18 cell lines identified to have changes suggestive of chromothripsis. The top panel for each cell line shows the copy number profile across the chromosome, the middle row shows the probability of a change in copy number state at each position and the lower panel shows the allelic ratio. Homozygous SNPs cluster at allelic ratios near 0 or 1, heterozygous SNPs cluster around 0.5. (T) Segmented copy number profiles with evidence of chromothripsis from representative samples in the ‘Tumorscape’ database from the Broad Institute (Beroukhim et al., 2010). [file mmc6.pdf]

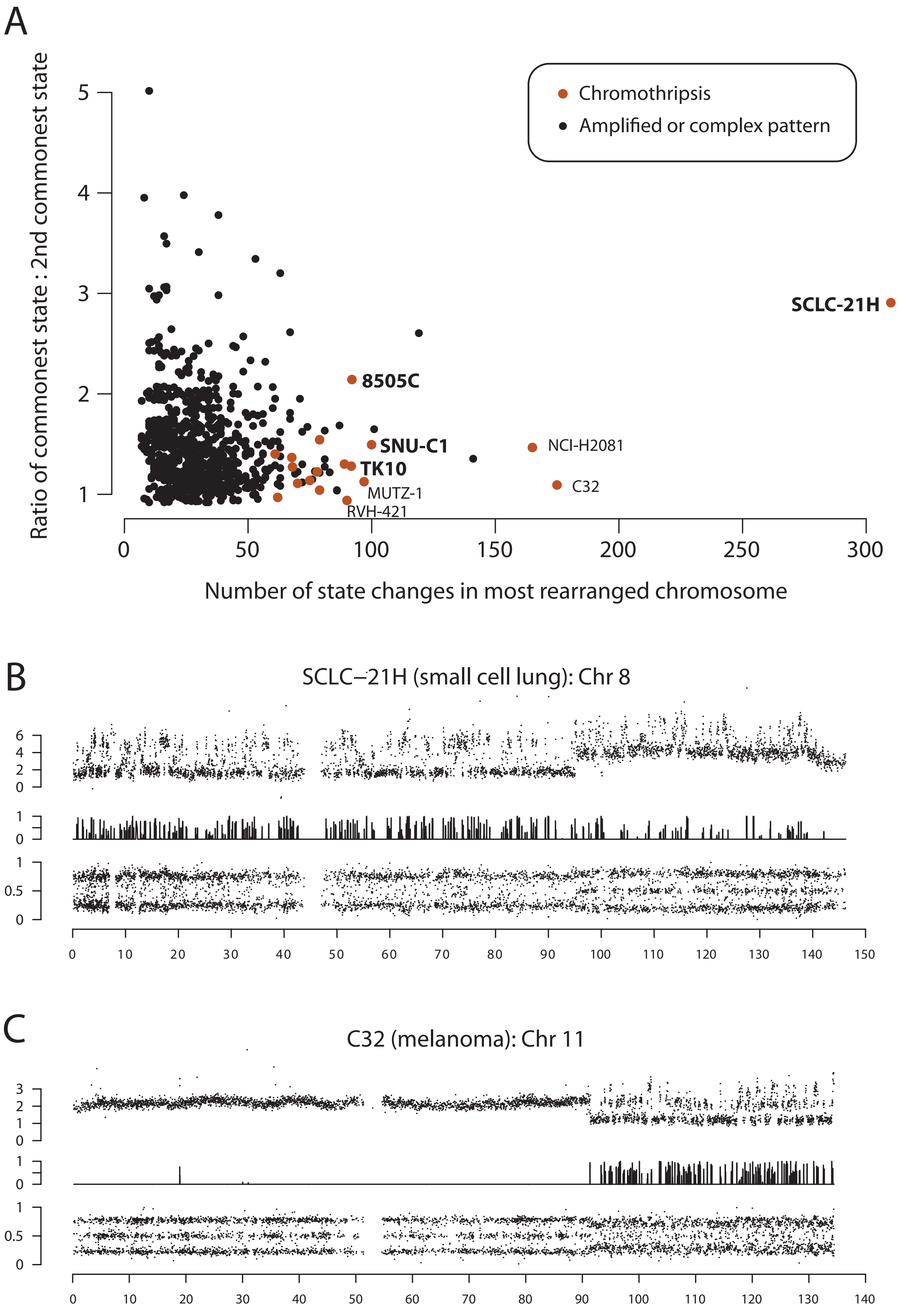

Supplementary figure S1, A-C

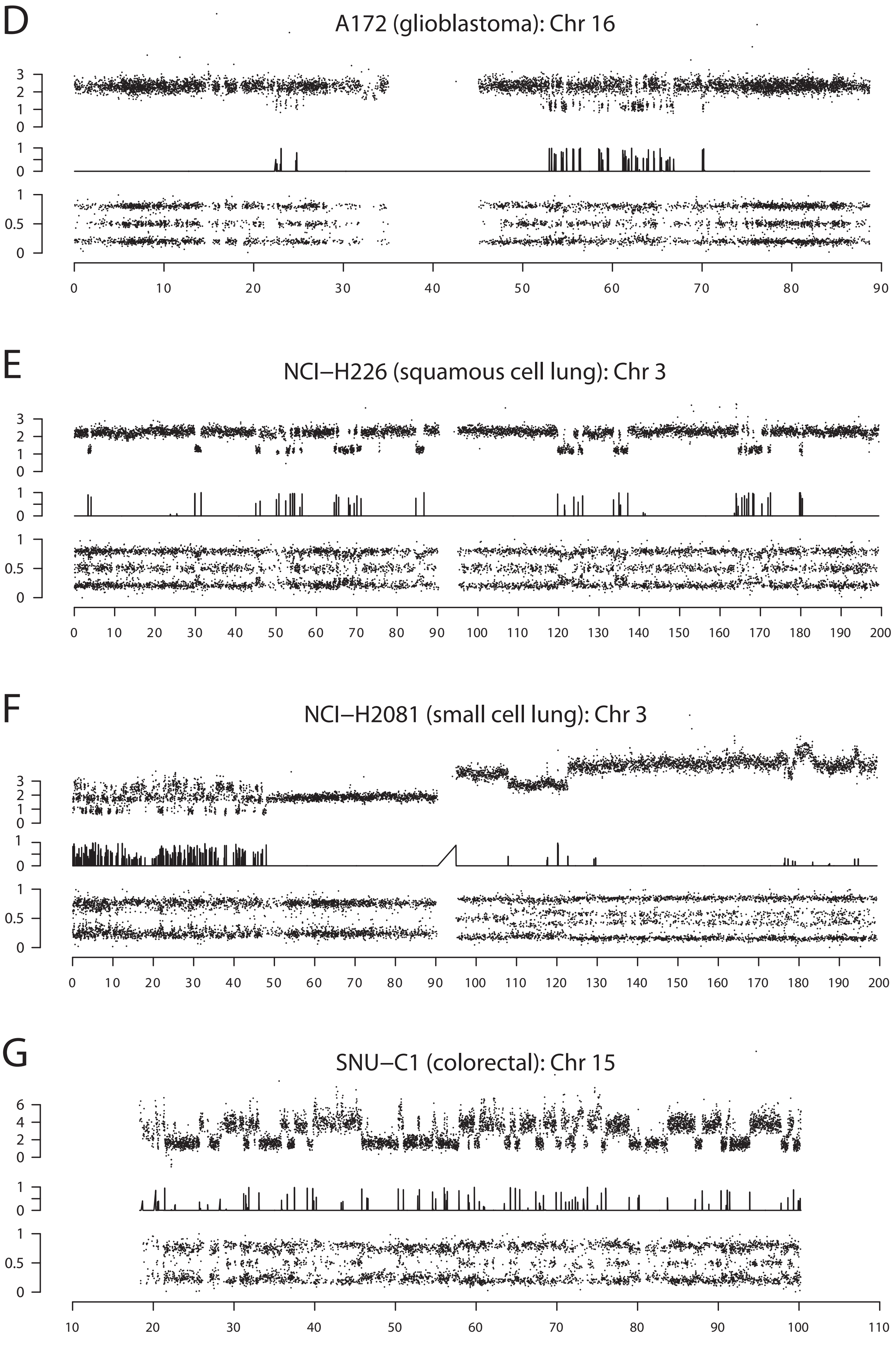

Supplementary figure S1, D-G

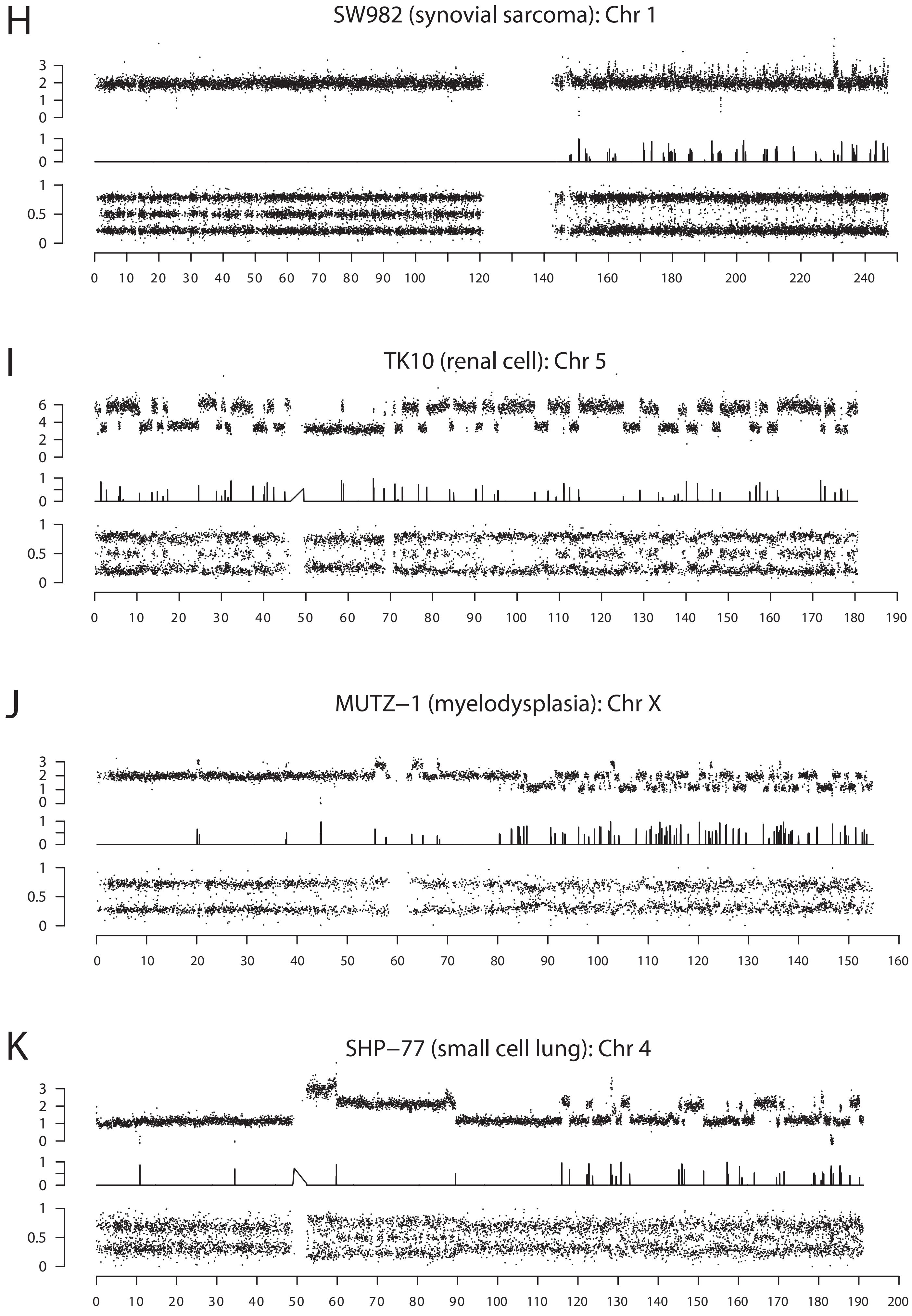

Supplementary figure S1, H-K

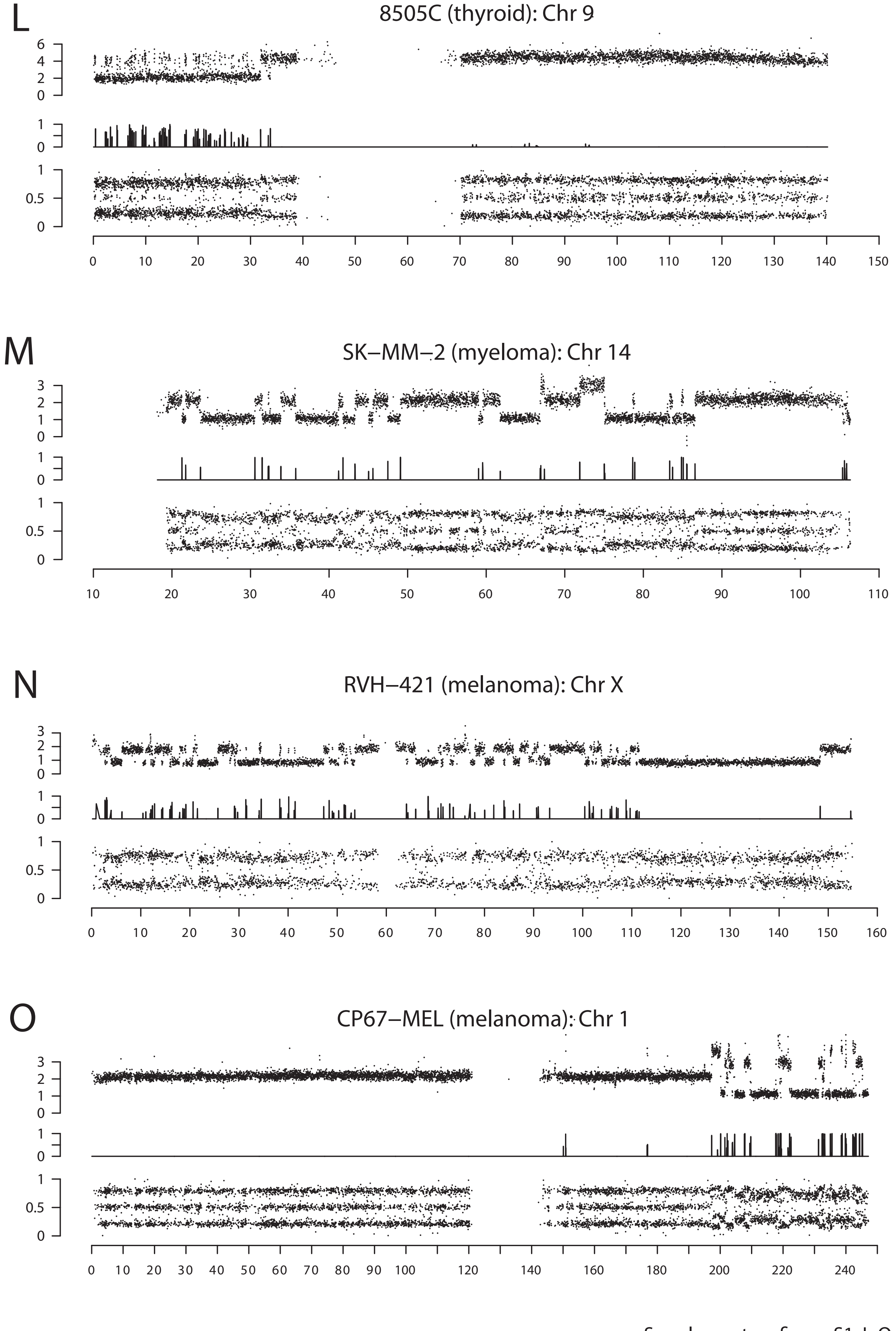

Supplementary figure S1, L-O

P

KYSE−450 (oesophageal): Chr 4

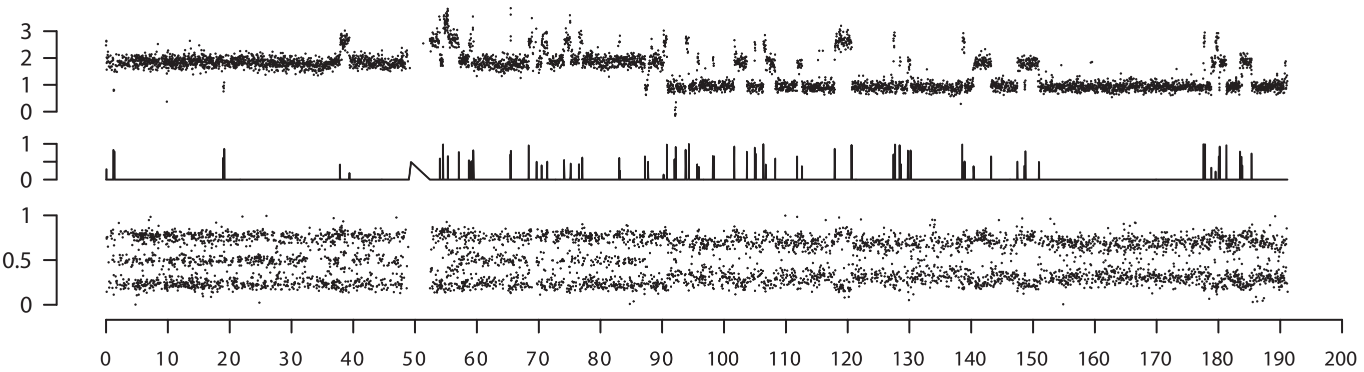

Q

HMV−II (melanoma): Chr 3

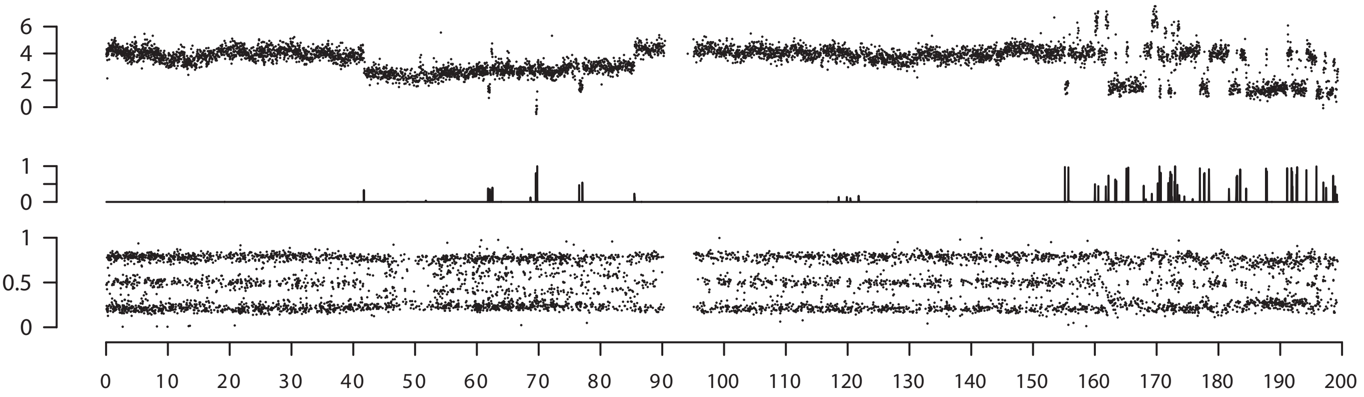

R

D−397MG (astrocytoma): Chr 7

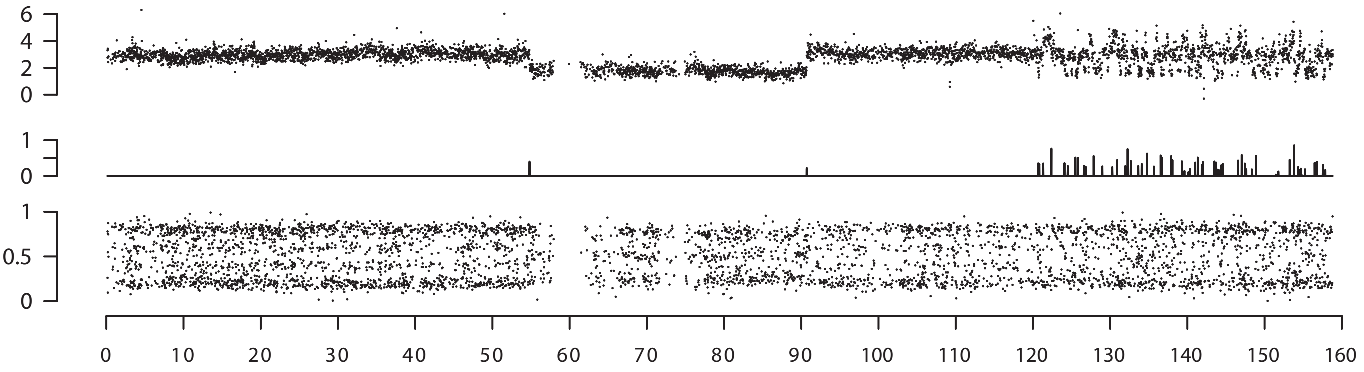

S

D−538MG (astrocytoma): Chr 3

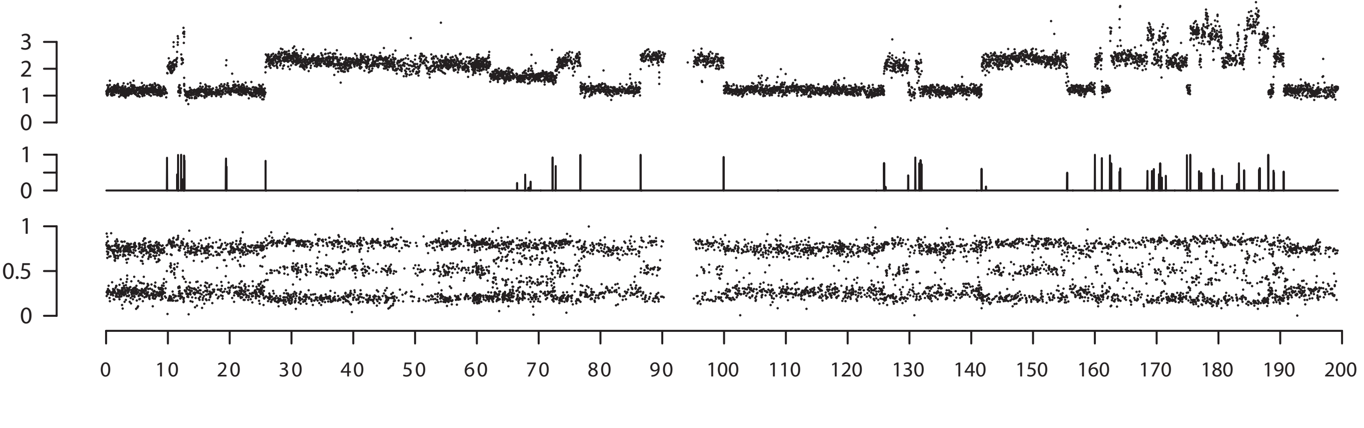

Supplementary figure S1, P-S

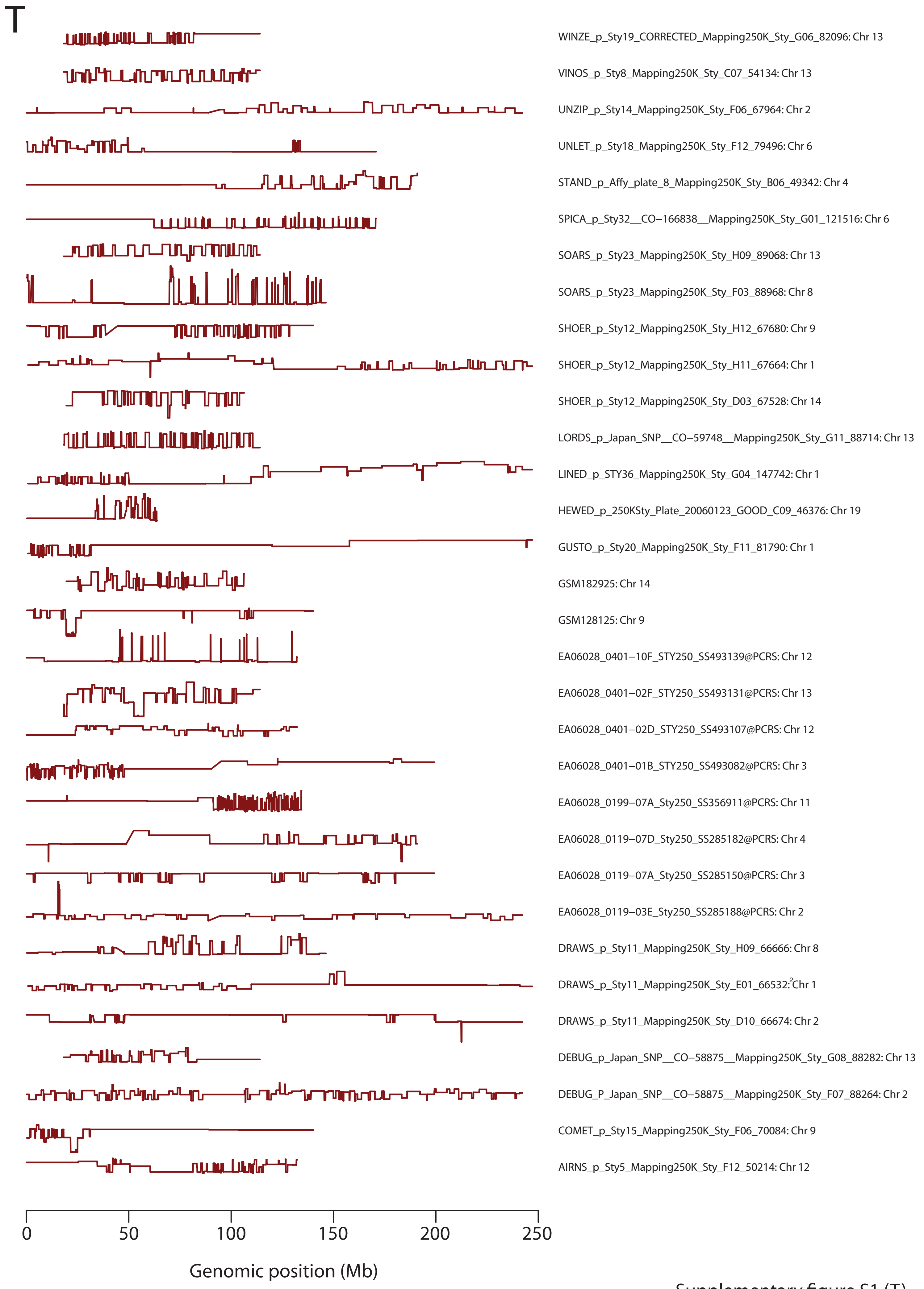

Supplementary figure S1 (T)
